# ASXL1 promotes adrenocortical carcinoma and is associated with chemoresistance to EDP regimen

**DOI:** 10.18632/aging.203534

**Published:** 2021-09-18

**Authors:** Liang Wang, Yinfeng Lyu, Yuqing Li, Kunping Li, Hui Wen, Chenchen Feng, Ning Li

**Affiliations:** 1 Department of Urology, Tianjin Medical University General Hospital, Tianjin 300052, P.R. China; 2 Department of Urology, Huashan Hospital, Fudan University, Shanghai 200040, P.R. China; 3 Department of Urology, Fourth Affiliated Hospital of China Medical University, Shenyang 100032, Liaoning Province, P.R. China

**Keywords:** adrenocortical carcinoma, ASXL1, chemoresistance

## Abstract

Adrenocortical carcinoma (ACC) is a rare but aggressive disease that lacks definitive treatment. We aim to evaluate role of ASXL1 in ACC and exploit its therapeutic merits therein. We performed *in silico* reproduction of datasets of the Cancer Genome Atlas (TCGA), GDSC (Genomics of Drug Sensitivity in Cancer) and Human Protein Atlas using platforms of cBioPortal, UALCAN, NET-GE, GSEA and GEPIA. Validation in ACC was performed in tissue, *in vitro* and *in vivo* using the NCI-H295R and SW-13 cells. ASXL1 was gained in over 50% of ACC cases with its mRNA overexpressed in DNA gained cases. ASXL1 overexpression was associated with recurrence and worsened prognosis in ACC. ASXL1 gain was associated with resistance to etoposide, doxorubicin and cisplatin (EDP). ASXL1 expression was positively correlated with FSCN1 expression. Targeting ASXL1 significantly impaired fitness of ACC cells, which could be in part rescued by FSCN1 overexpression. Targeting FSCN1 however could not rescue resistance to EDP induced by ASXL1 overexpression. Targeting ASXL1 sensitized ACC cells to EDP regimen but constitutive ASXL3 overexpression in SW-13 cells could induce resistance upon prolonged treatment. Functional gain of ASXL1 was common in ACC and exerted pro-tumorigenic and chemoresistance role. Targeting ASXL1 hold promise to ACC treatment.

## INTRODUCTION

Adrenocortical carcinoma (ACC) originated from adrenal cortex cells, as a rare neoplasm with annual incidence of 0.7~2 per million [1], is highly aggressive and is prone to metastasize. Lack of heralding symptoms often render patients with ACC diagnosed at a late stage. Mitotane, as the only FDA approved specific drug for ACC, together with surgery, chemo- and radio-therapy forms a multi-modality treatment scheme for ACC at present. However, for stage IV patients catalogued by traditional European Network for the Study of Adrenal Tumors (ENSAT) classified method, their 5-years survival could merely reach 6% - 13% [2]. In general, current treatment for stage III-IV ACC is disappointing.

Current clinical practice guidelines on the management of adrenocortical carcinoma was issued by European Society of Endocrinology (ESE) in 2018. In terms of drug treatment, based on the results of the FIRM-ACT trial, the guidelines recommend the utilization of mitotane +EDP (etoposide, Adriamycin, cisplatin combination chemotherapy) as the most effective remedy for advanced ACC patients who are not amenable to local treatment [3].

Recent high throughput sequencing studies revealed some genomic mutations remarks that be identified as “drive events” of ACC tumorigenesis including mutations in *IGF-2*, *TP53*, *CTNNB1*, *MEN1* and *PRKAR1A*. Somatic mutations and fusions of these genes mostly relate to continuous activation of WNT/β-catenin pathway which cause the overexpression of corresponding growth factors and inactivation of tumor suppressor *TP53*. In the meantime, there is a dearth of studies on the impacts of copy number variation (CNV) in ACC. Assie, Fernandez, Lerario et al. reported partial or complete deletion of chromosome 1p, 17p, 22p, 22q, 2q and 11q by comparative genomics via the microsatellite method loss of heterozygosity and allele imbalance in ACC [4–6]. However, insightful mechanistic analyses are still lacking.

The Cancer Genome Atlas (TCGA) project of ACC demonstrated unprecedented genomic and genetic panorama of the rare cancer which substantially promoted researches on ACC from bench to bedside [7]. However, many copy number variance (CNV) in ACC revealed in TCGA still lacked insightful functional investigation. In the current study, we set off to evaluate role of 20q gain in ACC and identified Additional sex combs like transcriptional regulator 1 (ASXL1) as target gene that could mediate chemoresistance with the aim of deepening the understanding of biology of ACC.

## MATERIALS AND METHODS

### *In silico* analysis

TCGA dataset was used to examine expression of ASXLs in ACC. Using the cBioPortal platform, we reproduced ACC dataset of TCGA. Gain of function was defined as over-expression whose z-score was of “Exp >= 2” and copy number gain or amplification. we plotted cases with gain of function of ASXLs using the OncoPrint function of cBioPortal. Enriched genes at mutation and expression level in cases with gain of function of ASXL1 was generated at cBioPortal and were then submitted to NET-GE analytical platform for functional annotation. Survival analysis and correlation of gene expression were generated using cBioPortal and GEPIA, respectively.

### Immunohistochemistry (IHC)

An in-house tissue microarray containing 54 paraffinized sections of ACC sample was used. All samples were surgically removed ranging from 1990 to 2019 from authors’ institutes. Informed consent was acquired in all patients and the study was approved by local ethics committee. Briefly, chip was first sliced, mounted, and subsequently deparaffinized. Tumors on xenograft models were prepared in the same manner. Sections were dehydrated with alcohol and hydrogen peroxide was applied for blockade. The sodium citrate buffer solution was prepared for antigen retrieval with microwave. The non-fat milk was applied for non-specific antigen blockade. Primary antibodies against ASXL1 (ThermoFisher, PA5-71475, 1:50) and ASXL3 (Novus Biologicals, NBP2-14791, 1:50) were applied overnight. Secondary antibody and DAB was subsequently applied and the section was counter-stained with hematoxylin. Production intensity and extensity of staining was first calculated. Intensity score ranged from 0 to 3 representing from no staining to brown. Extensity score was as follows: 0 for negative, 1 for 1-25% cells stained, 2 for 26%-50%, 3 for 51-75% and 4 for 76%-100%. A final score of 0-4, 5-8 and 9-12 corresponded to IHC score of 0, 1 and 2 respectively. Sections were all de-identified and only information on cancer subtype, TNM stage, and tumor grade were available. The whole experiment conformed to Declaration of Helsinki.

### Cell line and RNA interference

The NCI-H295R and SW-13 cells were obtained from the National Experimental Cell Resource Sharing Platform of China. Cell lines were cultured with RPMI 1640 medium supplemented 10% bovine serum. shRNAs targeting ASXL1 and FSCN1 were selected from GPP (https://portals.broadinstitute.org/gpp/public/) as follows: TRCN0000379665 (shRNA#1) and TRCN0000285358 (shRNA#2) for ASXL1; TRCN0000123042 and TRCN0000123040 for ASXL3. A standard non-lipofectamine transfection protocol was followed and puromycin-resistant clone was selected. Green-fluorescent protein (GFP) was integrated for efficacy determination. Treatment of EDP was performed as previously reported [8].

### RNA isolation and quantitative PCR (qPCR)

We used TRIzol reagent (Invitrogen) to extract the total RNA of the cells according to the instructions of the manufacturer. PrimeScrip RT Master Mix (TakaRa) was employed to perform reverse transcription reactions of RNA samples. For determining the expression levels of cDNA, SYBR Premix ExTaq II (TaKaRa) was used to conduct quantitative real-time polymerase chain reaction (qRT-PCR) analyses according to manufacturer’s protocols. The internal control in this experiment was GAPDH. Ct method was used to calculate the relative abundance of mRNA after normalization. Primers used were as follows: ASXL1, Forward 5’-CGCGCCTGGTATTAGAAAACT-3’; Reverse 5’- GCATCCTTCTTGAGCGTGAAAAG-3’; FSCN1, Forward 5’- CCAGGGTATGGACCTGTCTG-3’; Reverse 5’- GTGTGGGTACGGAAGGCAC-3’.

### Luciferase activity assay

NCI-H295R cells were co-transfected with promoter firefly luciferase of target genes and plasmids of gene of interest using Lipofectamine Reagent (Invitrogen). Thirty-six hours later, luciferase activity was measured using the Dual-Luciferase Reporter Assay System (Promega) according to the manufacturer’s protocol. Luciferase activity was normalized to Renilla luciferase activity.

### Transwell and wound-healing assays

For transwell migration assay, with polycarbonate membrane as separation, DMEM and 10% FBS served as nutrient solution in outer chamber, while 7 x 10^4^ tumor cells per well were put into the inner chamber. After 16 hours, the migrated cells were stained by crystal violet and counted by microscope. For invasion assay, transwell inserts (Costar) coated with Matrigel (BD Biosciences)/fibronectin (BD Biosciences) was utilized. To perform the wound healing assay, cell suspension was cultured 16-24 hours as monolayer cells. Scratch the cells with the head of pipetting gun and add the 5-Fu solution incubating for 24 hours, and change the 10% FBS for 24 hours. Inverted microscope was used for observation and image capture.

### Xenograft murine model

Tumor cells were cultured in DMEM medium. Extract 100 μl of the mixed cell suspension with 1 ml syringe and inoculated subcutaneously on the right hind limb of the right back of the nude mice. On the 12th day of inoculation, the tumor volume of all nude mice was > 100mm^3^. The spirit, diet, defecation and activity of the nude mice were observed daily. Application of EDP-M regimen was strictly according to previous report [9]. The mass of the transplanted tumor was weighed and the long diameter (A) and short diameter (B) of the transplanted tumor were measured with a vernier caliper every 3 days from the 3rd day of inoculation. The mean volume of the transplanted tumor was calculated according to the volume formula V=1/2 (A×B^2^), and the average value was obtained and the curve of tumor growth was plotted. On the 60^th^ day after inoculation, the nude mice were sacrificed and the tumor was removed, and the morphology, texture and activity of the transplanted tumor were observed. Endpoints included tumor reaching cut-off volume of 2000 mm^3^ or necrosis. Animal experiments were approved by Department of Laboratory Animal Science of Fudan University.

### Statistical analyses

Statistical analysis for *in silico* studies were automatically performed with the platforms used, as aforementioned. Statistical analysis for *in vitro* assays and *in vivo* experiments were performed using the Prism Graphpad 9.0 for Mac. Comparisons between two groups were studied using the Mann-Whitney test for non-parametric variants and using the Student’s t test for parametric variants. IC50 for drug treatment was interpolated and fitted with sigmoidal curve. The survival data was presented using the Kaplan-Meier curve and compared using the Log-rank test. The P value of <. 05 was accepted as significant.

## RESULTS

**Figure 1:**
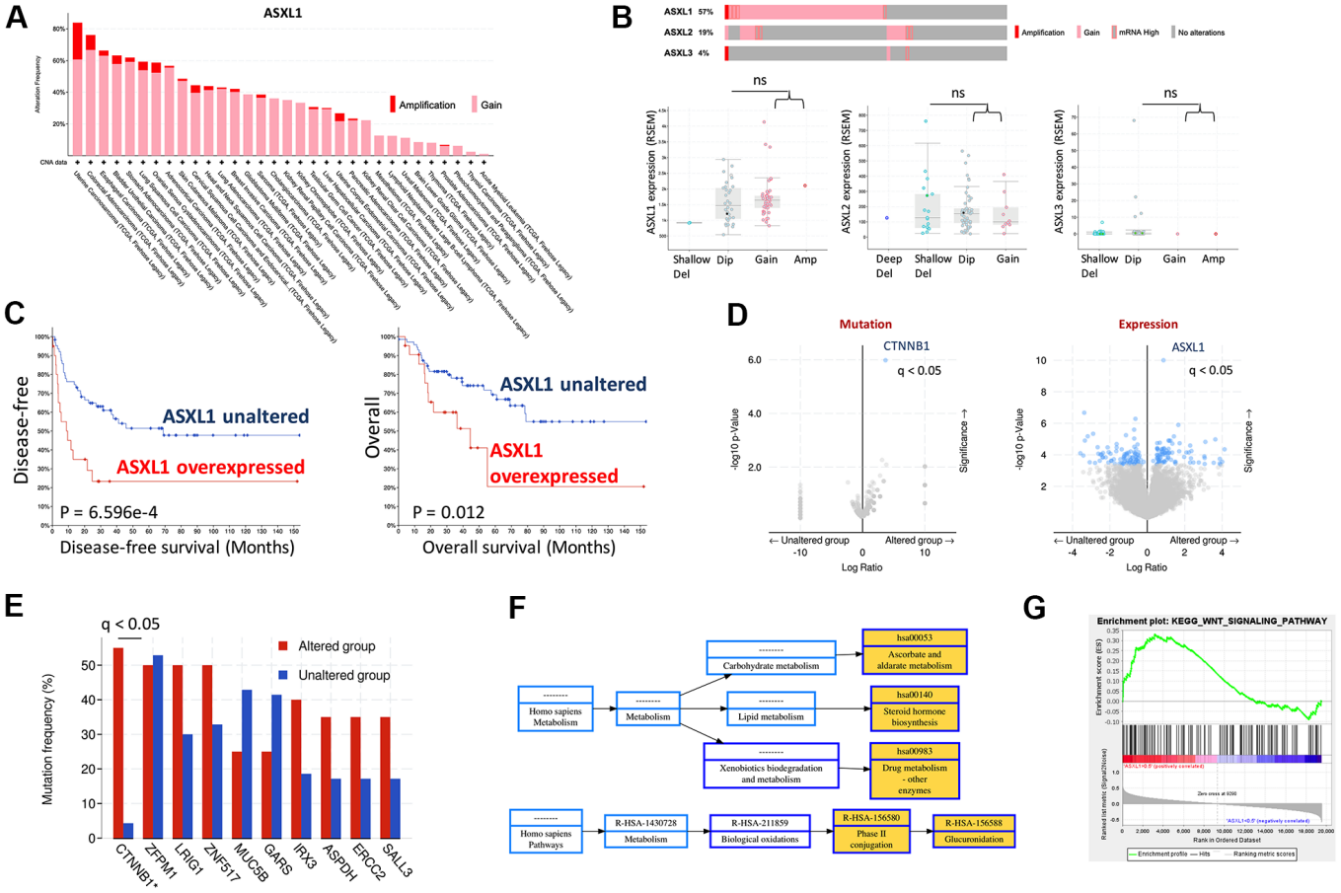
**Gain of function of ASXL1 was common in adrenocortical carcinoma (ACC).** (**A**) Reproduced from TCGA pan-cancer cohorts (Firehose) and ACC cohort (N = 75), shown were Gain and amplification of ASXL1 across cancer types; (**B**) Cases with gain of function of ASXL1 (N = 51), ASXL2 (N = 17) and ASXL3 (N = 3) and box plot of expressions of ASXL1, ASXL2 and ASXL3 against copy number alteration; (**C**) Kaplan-Meier plots of overall and disease-free survival in ACC patients with or without ASXL1 overexpression, compared by the log-rank test; (**D**) Volcano plot of significantly enriched mutation and expression of genes in ACC cases with gain of function of ASXL1; (**E**) Bar figure showing frequency of gene mutation in ASXL1-altered (gain of function) and -unaltered group; (**F**) Functional annotation by NET-GE of genes enriched in (**D**) using KEGG; (**G**) GSEA analysis of genes enriched in (**D**) (ns = not significant; q value denoting false discovery rate, *P < 0.05; **P <0.01).

CNV of ASXL1 was first queried across cancer types and ACC was amongst top cancers that harbored ASXL1 gain (Figure 1A). We then focused on TCGA ACC cohort and found gain-of-function was the predominant alteration in all 3 ASXLs (Figure 1A). Of note, gain of ASXL1 occurred in ~57% of ACC cases (Figure 1B). We further plotted mRNA expression against copy number in all 3 ACXLs and found only ASXL1 showed increased mRNA expression along with increased copy number, indicating functional output of the gene (Figure 1B). Overexpression of AXSL1 conferred both worsened overall and progression-free survival in ACC patients (Figure 1C). Enrichment analysis showed CTNNB1 mutation, one of the established driver events in ACC, was significantly enriched in ASXL1-gained cases (Figure 1D, 1E). Gene expression enrichment analysis also showed significantly enriched drug metabolism pathway (Figure 1F) and WNT signaling (Figure 1G).

**Figure 2:**
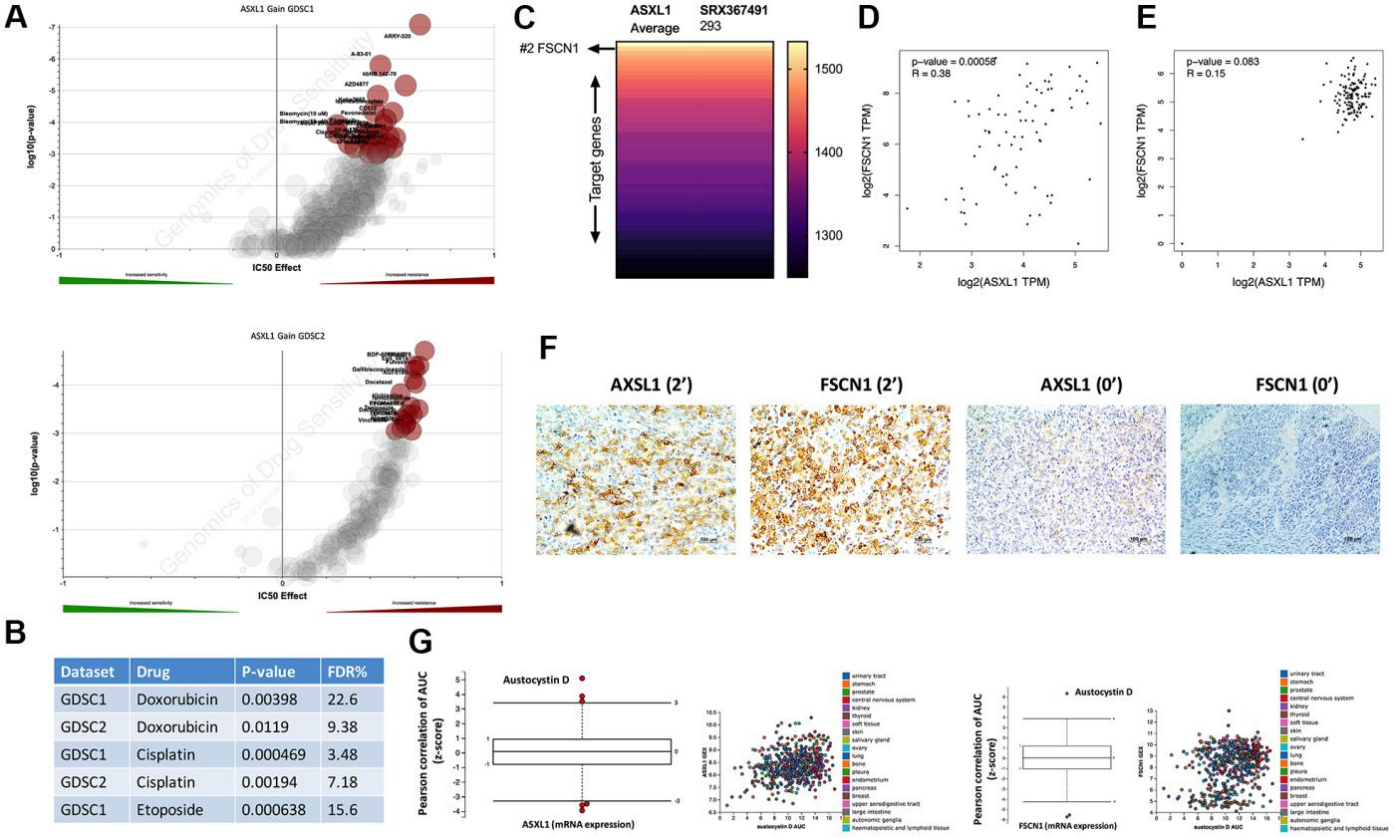
**Expressions of ASXL1 and FSCN1 was positively correlated.** Reproduced from the GDSC dataset, shown were volcano plots of drug sensitivity of gain of cytoband encompassing ASXL1 in pan-cancer cell lines from (**A**) GDSC1 and GDSC2 repositories and (**B**) retrieved P values for Doxorubicin, Etoposide and Cisplatin; (**C**) Reproduced from ChIP-Atlas, shown was heatmap ranked by binding score of ASXL1 from high to low in different model cells (human 293 cells), each row representing one gene; (**D**) Reproduced from the TCGA adrenocortical carcinoma (ACC) dataset, shown was expression correlation between ASXL1 and FSCN1 in ACC samples; (**E**) Reproduced from the GTEx dataset, shown was expression correlation between ASXL1 and FSCN1 in normal adrenal samples; (**F**) Representative IHC image of ASXL1 and FSCN1 staining in ACC samples with numbers indicating IHC score; (**G**) Reproduced from The Cancer Therapeutics Response Portal, shown were box plots of correlation coefficients between area under control and gene expression in cancer cells.

We then pursued drug metabolism function of ASXL1 gain. By querying cytoband that encompassed ASXL1 that was amplified in GDSC dataset, we showed that ASXL1 gain was associated with significant resistance to a variety of compounds in both GDSC1 and GDSC2 pools (Figure 2A). Amongst the compounds were three chemo-agents (EDP) that consisted of the first line chemotherapy of ACC (Figure 2B). ASXL1 functioned in chromatin remodeling and regulates the expression of many genes. To identify target gene(s) of ASXL1 we performed analysis of the ChIP-Atlas and found FSCN1 ranking top 2 in binding affinity to ASXL1 (Figure 2C). Of note, FSCN1 was reported to play important pro-tumorigenic role in ACC [10]. Reproduction of TCGA ACC cohort showed significant linear positive correlation between expressions of ASXL1 and FSCN1 (Figure 2D). Interestingly, this correlation did not exist in normal adrenal tissue (Figure 2E). Analysis of clinicopathological parameters in TCGA ACC cohort showed ASXL1 expression was significantly higher in tumors with incomplete removal, with marginal significance in tumors of higher stage and progressed disease upon adjuvant treatment (Supplementary Table 1). In our IHC cohort, we validated linear correlation of expression of ASXL1 and FSCN1 using IHC scores (r = 0.823, P <0.001) (Figure 2F) and showed AXSL1 expression being significantly higher in tumors with advanced staging and in nodal positive cases with marginal significance (Table 1). We then used an alternative strategy to validate ASXL1/FSCN1 axis by plotting drug sensitivity profile against expressions of ASXL1 and of FSCN1 in the CTRP platform that incorporated multiple datasets. We showed austocystin D being the top hit in both queries (Figure 2G).

**Table 1:** Association of ASXL1 expression and clinicopathological parameters in our in-house cohort.

	**IHC score (non-parametric)**		
	***N***	Median	SE
**Clinical Stage**			
I	14	1	0.256776296
II	20	2	0.160591014
III	7	2	0.260820265
IV	13	2	0.307692308
*P value*		0.005	
**Atypical Mitotic Figures**			
Present	25	2	0.2212088
Absent	29	2	0.160155352
*P value*		0.417	
**Clinical Status 3 Mo Post-Op**			
Present	17	2	0.260588899
Absent	37	2	0.15525845
*P value*		0.611	
**Lymph node**			
N0	42	2	0.160065041
N1	12	2	0.166666667
*P value*		0.074	
**T stage**			
T1	14	1	0.256776296
T2	25	2	0.144683563
T3	6	2	0.25819889
T4	9	2	0.323941772
*P value*		0.0002	

**Figure 3:**
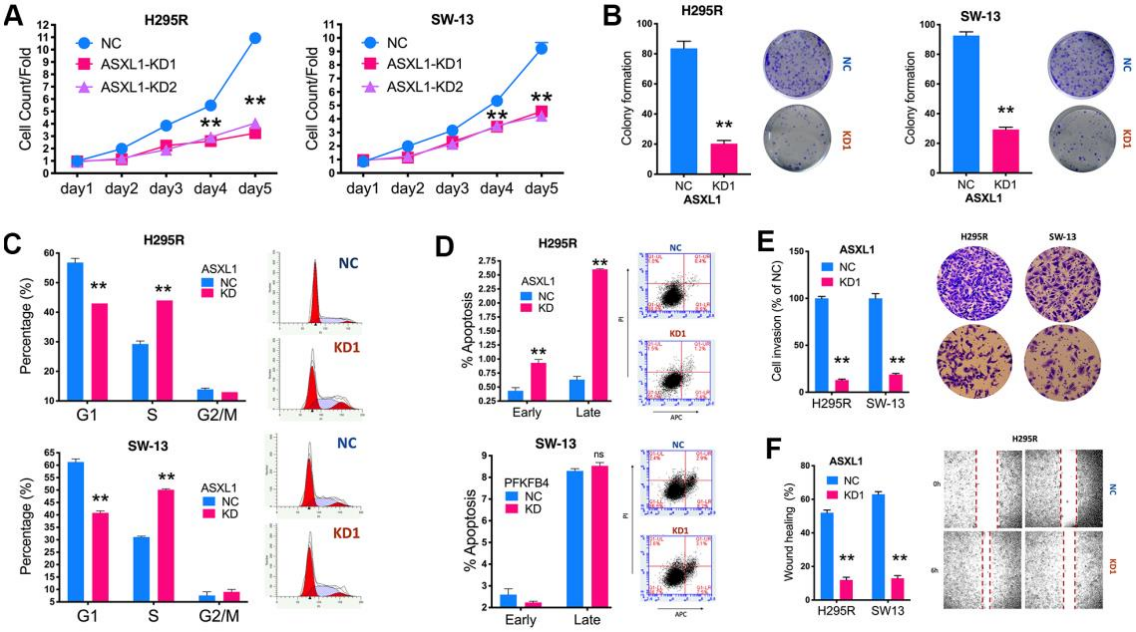
**Silencing of ASXL1 decreased fitness of adrenocortical carcinoma (ACC) cells.** (**A**) Cell count detected using CCK-8 in ACC cell lines with ASXL1-knockdown (KD) by shRNA#1 and shRNA#2 (KD1 and KD2) or negative control (NC); (**B**) Colony formation in ACC cell lines with ASXL1 silencing or control); Flow cytometry used to detect (**C**) cell cycle profile and (**D**) apoptosis in ACC cells with ASXL1-KD or NC; (**E**) Transwell assays used to detect cell invasion with Matrigel in ACC cells with ASXL1-KD or NC, captured at 100×; (**F**) Wound healing assay in ACC cells with ASXL1-KD or NC (**P < 0.01).

**Figure 4:**
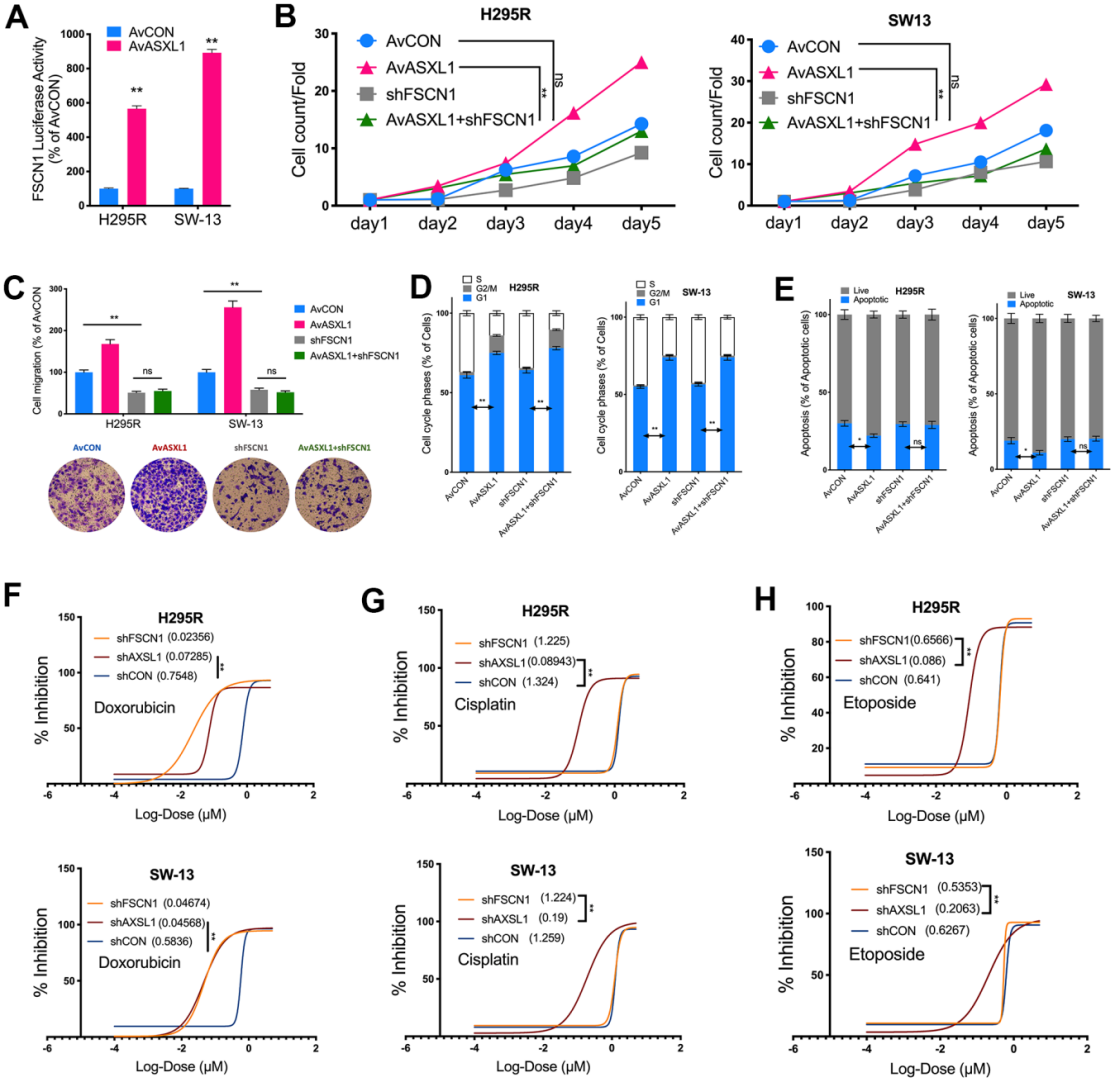
**Silencing ASXL1 sensitized ACC cells to etoposide, doxorubicin and cisplatin (EDP) regimen.** (**A**) Relative firefly-luciferase activity of FSCN1 in NCI-H295R cells where Firefly luciferase activity was normalized to Renilla luciferase activity for all samples to yield relative luciferase activity upon ASXL1 silencing or control; (**A**, **B**) Cell count detected using CCK-8 in ACC cell lines with ASXL1 overexpression by adenovirus (Av) or control (AvCON) and/or with FSCN1 silencing by shRNA; (**C**) Transwell assays used to detect cell invasion with Matrigel in ACC cells with ASXL1 overexpression by adenovirus (Av) and/or with FSCN1 silencing by shRNA, captured at 100×; Flow cytometry used to detect (**D**) cell cycle profile and (**E**) apoptosis in ACC cells with ASXL1 overexpression by adenovirus (Av) and/or with FSCN1 silencing by shRNA; Dose-response curves of (**F**) Doxorubicin, (**G**) Cisplatin and (**H**) Etoposide in ACC cells with ASXL1 or FSCN1 silencing by shRNA or control (shCON), with IC50 (μmol) in parentheses (ns = not significant; *P < 0.05; **P < 0.01).

We then evaluated effect of ASXL1 *in vitro* in 2 ACC cell lines. Silencing of ASXL1 significantly inhibited proliferation in both cell lines (Figure 3A). ASXL1-KD also significantly inhibited colony formation (Figure 3B). Silencing of ASXL1 significantly lowered population in G1phase and increased population in S phase in both cell lines (Figure 3C). ASXL1-KD also led to increased early and late apoptosis in NCI-H295R cell but showed no effect on apoptosis in SW-13 cells (Figure 3D). For cell motility, we showed that ASXL1-KD significantly decreased cell invasion (Figure 3E) and wound healing (Figure 3F) in both cell lines. In the rescue assays, we first found that transcriptional activity being significantly increased in both cell lines with ASXL1-OE (Figure 4A). FSCN1 silencing restored promotion of proliferation and cell migration by ASXL1-OE in both ACC cell lines (Figure 4B, 4C). However, FSCN1 silencing did not alter cell cycle profile and apoptosis interfered by ASXL1-OE in either cell line (Figure 4D, 4E). We then examined drug sensitivity profile of EDP. We found both ASXL1 and FSCN1 silencing decreased IC50 of doxorubicin in both ACC cell lines (Figure 4F). Nonetheless, only ASXL1-KD but not FSCN1-KD could cold lower IC50 of cisplatin (Figure 4G). Similarly, FSCN1-KD showed minimal effect on sensitivity of etoposide whereas ASXL1-KD increased sensitivity (Figure 4H).

**Figure 5:**
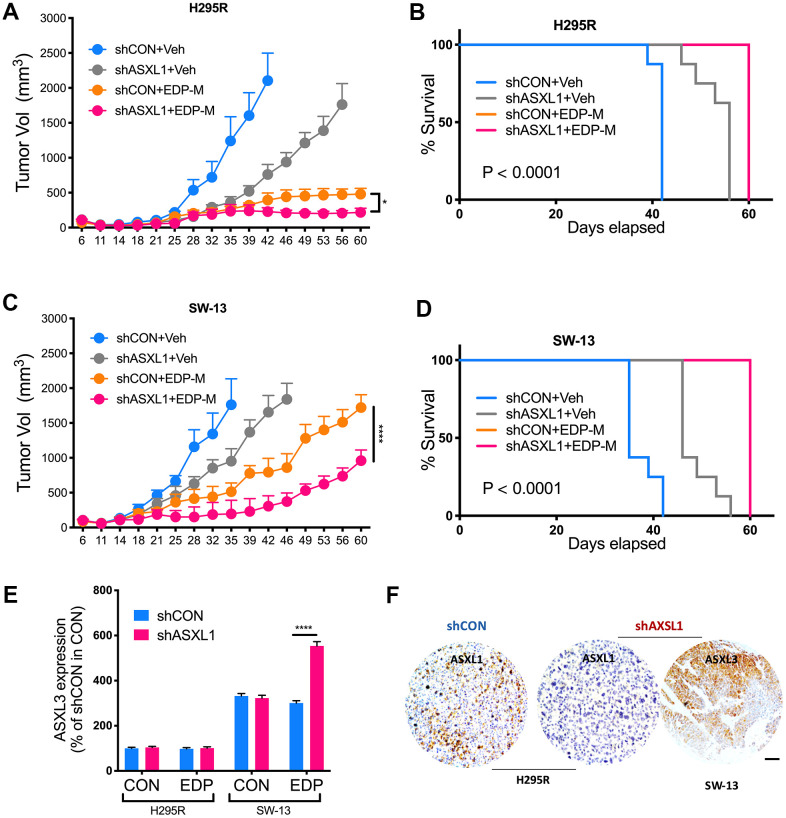
**ASXL1 silencing sensitized ACC cells to etoposide, doxorubicin, cisplatin and mitotane (EDP-M) regimen.** Xenograft murine models consisting of 8 male BALB/c nude mice per group undergoing EDP-M regimen () or vehicle control (Veh) with subcutaneous implanted with (**A**) NCI-H295R cells with or without ASXL1 silencing under left flank with tumor growth monitored over 60-day period and tumor size of < 2000 mm^3^ as endpoint, and (**B**) Kaplan-Meier curves of survival of mice; and with (**C**) SW-13 cells with or without ASXL1 silencing under left flank with tumor growth monitored over 60-day period and tumor size of < 2000 mm^3^ as endpoint, and (**D**) Kaplan-Meier curves of survival of mice; (**E**) Quantitative PCR showing expression of ASXL3 in ACC cell lines treated with EDP regimen; (**F**) Representative image of immunohistochemical staining of ASXL1 and ASXL3 in harvested tumors from xenograft models (scale bar = 100 μm, *P < 0.05; **P < 0.01; ***P < 0.001; ****P < 0.0001).

We next evaluated role of ASXL1 *in vivo*. Both silencing of ASXL1 and EDP-M regimen significantly inhibited tumor growth of NCI-H295R cells, whereas addition of ASXL-KD further inhibited tumor growth (Figure 5A). Survival analysis showed significantly prolonged survival in group with ASXL1-KD and EDP-M (Figure 5B). Interestingly, whereas tumors in SW-13 xenograft models grew at a similar rate to NCI-H295R, SW-13 tumors showed trend towards acquired insensitivity upon prolonged treatment starting on day 39 (Figure 5C). Combination of EDP-M and ASXL-KD also showed similar trend although the combination group showed most potent inhibition (Figure 5C, 5D). As ASXL3 was an important paralog of ASXL1 [11] and ASXL3 was constitutively overexpressed in SW-13 cells, we evaluated ASXL3 expression in both cells and found EDP could only induced increased ASXL3 expression in SW-13 cells (Figure 5E). IHC in harvested tumors also showed decreased ASXL1 expression in ASXL1-KD group and strong ASXL3 expression in SW-13 cells (Figure 5F).

## DISCUSSION

Stage IV ACC confers a 5-year overall survival of 0% and novel treatment modality is at urgent need [12]. Our study highlights both the protumorigenic role and chemoresistance of ASXL1 gain in ACC. Normal ASXL1 plays a role in embryonic development. ASXL1 mutations are observed primarily in myelodysplastic syndromes, but they are also observed in colorectal and endometrial cancer [13, 14]. ASXL1 is altered in 4.42% of all cancers with colon adenocarcinoma, lung adenocarcinoma, breast invasive ductal carcinoma, acute myeloid leukemia, and myelodysplastic syndromes. The most common alterations in ASXL1 is mutation (2.62%) followed by amplification (0.67%). As a driver event in several cancers, ASXL1 status has been recognized as an inclusion criteria in cancer trials targeting hematological malignancies such as acute myeloid leukemia, myelodysplastic syndromes, acute myeloid leukemia arising from previous myelodysplastic syndrome, chronic myelomonocytic leukemia, and secondary acute myeloid leukemia [15].

Our study has been the first to identify the role of ASXL1 in ACC. Though ASXL1 is solely amplified in 1 case in ACC, CN gain occurred in over half of cases and the increased mRNA expression following CN gain is indicative of gain of function of the gene. This is also consolidated by its prognostic value at mRNA level. Of note, TCGA cohort shows ASXL1 is associated with incomplete resection and our cohort indicates ASXL1 is associated with advanced stage. Both findings suggest that ASXL1 gain confers growth advantage to treatment-naïve ACC.

Being the first and only phase III randomized controlled trial, the FIRM-ACT trial provided by far the most solid evidence for chemotherapy regimen. The EDP-M regimen is now recognized as the first line therapy for advanced ACC [16]. Immune checkpoint inhibitors (ICIs) that reinforced last defense in many solid tumors only show moderate effect in ACC and are currently used under salvage and trial settings [17]. In fact, most trials trying to transplant agents effective in other cancers into ACC yield frustrating outcomes [18]. The nature of multi-resistance and rare disease renders drug development stumbling over decades.

Genetic landscape of ACC revealed by TGCA triggers various translational studies on ACC and our findings in the current study also takes advantage of the CNV data of TCGA. We for the first time report association between EDP sensitivity and CN of a certain gene. Gain of function in both ASXL1 and FSCN1 plays critical role in mediating resistance. Interestingly, most positive findings in NCI-H295R cells has also been observed in SW-13 cells that is now considered to originate from small cell carcinoma of adrenal gland. Furthermore, overexpression ASXL3 in SW-13 cells suggests that crosstalk may exist in ASXL family to facilitate resistance. Nonetheless, we find that gain of function of ASXL3 occurs in only 4% of ACC cases, further supporting different cell context of SW-13 and NCI-H295R. The interplay between FSCN1 and ASXL1 has not been reported before. However, overactive FSCN1 has been reported to be pro-tumorigenic in a variety of cancers [19–21]. Detailed mechanism of how FSCN1 mediate ASXL1 expression in the presence of ASXL1 copy number gain, another driver incentive, would be interesting.

Our study also has limitations. Although ASXL1 appears to be a promising target in ACC, we failed to test compound(s) that target ASXL1. Using gene-compound sensitivity dataset, we have identified austocystin D as a potential agent. However, the compound is not commercially available. Austocystin D is an organic heteropentacyclic compound isolated from *Aspergillus* and *Aspergillus ustus* and has been identified as a potent cytotoxic agent with *in vivo* antitumor activity and selectivity for cells expressing the multidrug resistance transporter MDR1, which was a characteristic of ACC as well [22–24]. Selective cytotoxic action of austocystin D arises from its selective activation by cytochrome P450 (CYP) enzymes in specific cancer cell lines, leading to induction of DNA damage in cells and *in vitro* [25]. The pattern of cytotoxicity of austocystin D was distinct from doxorubicin and etoposide putting austocystin D fairly promising in overcoming chemoresistance. Of note, as mitotane is a potent inducer of CYP3A4 activity which may have reduced the blood levels of doxorubicin and etoposide, both of which being metabolized by CYP3A4 [26, 27], monotherapy of austocystin D may in theory overcome this drawback. Production of austocystin D is now in progress by our group according to reported protocol and we look forward to testing the compound in ACC. Recent studies report that ACC cells are highly susceptible to ferroptosis, potentiating novel drug combination in this aggressive disease [28–30].

To sum up, we have shown that ASXL1 gain is common in ACC. Gain of function of ASXL1 confers worsened prognosis and promotes tumor growth of ACC. ASXL1 overexpression also induces chemoresistance to EDP regimen. Targeting ASXL1 hold promise to combat this rare but aggressive disease.

## Supplementary Material

Supplementary Table 1
