# Correction for: ASXL1 promotes adrenocortical carcinoma and is associated with chemoresistance to EDP regimen

**DOI:** 10.18632/aging.205255

**Published:** 2023-10-30

**Authors:** Liang Wang, Yinfeng Lyu, Yuqing Li, Kunping Li, Hui Wen, Chenchen Feng, Ning Li

**Affiliations:** 1 Department of Urology, Tianjin Medical University General Hospital, Tianjin 300052, P.R. China; 2 Department of Urology, Huashan Hospital, Fudan University, Shanghai 200040, P.R. China; 3 Department of Urology, Fourth Affiliated Hospital of China Medical University, Shenyang 100032, Liaoning Province, P.R. China

**Keywords:** adrenocortical carcinoma, ASXL1, chemoresistance

**This article has been corrected:** The authors found an error in **Figure 3B**. During assembly of the figure, incorrect images of colonies formed by ACC cell lines in which ASXL1 was silenced by shRNA#1 (KD1) were used. The authors prepared a new **Figure 3** using representative images from the original experiments. This correction has no impact on the main conclusion. The authors would like to apologize for any inconvenience caused.

New **Figure 3** is presented below.

**Figure 3:**
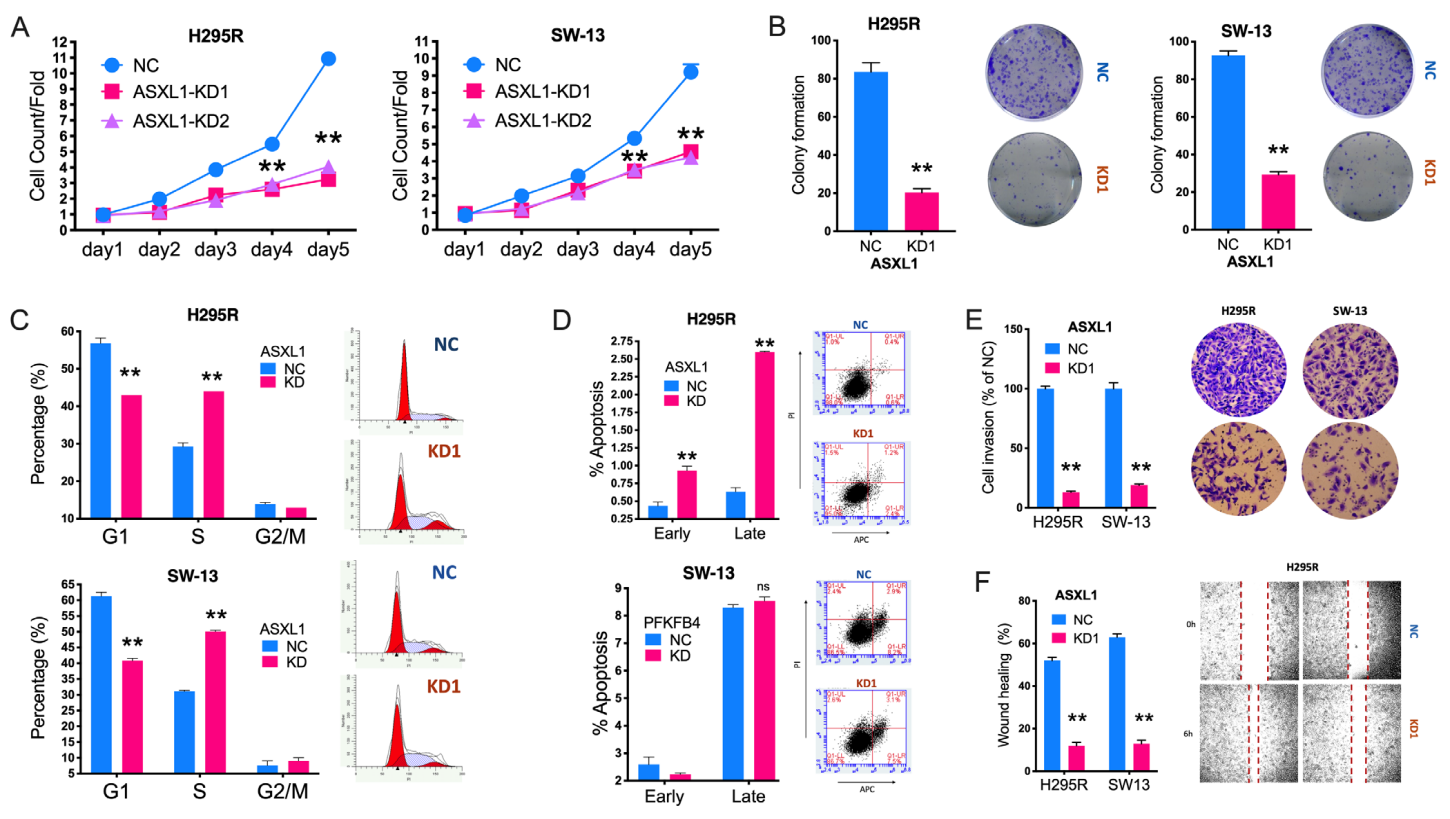
**Silencing of ASXL1 decreased fitness of adrenocortical carcinoma (ACC) cells. **(**A**) Cell count detected using CCK-8 in ACC cell lines with ASXL1-knockdown (KD) by shRNA#1 and shRNA#2 (KD1 and KD2) or negative control (NC); (**B**) Colony formation in ACC cell lines with ASXL1 silencing or control; Flow cytometry used to detect (**C**) cell cycle profile and (**D**) apoptosis in ACC cells with ASXL1-KD or NC; (**E**) Transwell assays used to detect cell invasion with Matrigel in ACC cells with ASXL1-KD or NC, captured at 100×; (**F**) Wound healing assay in ACC cells with ASXL1-KD or NC (**P < 0.01).

