# Supplementary material for: ASXL1 promotes adrenocortical carcinoma and is associated with chemoresistance to EDP regimen
Source: Aging (Albany NY). 2021 Sep 18;13(18):22286–97. doi: 10.18632/aging.203534 (PMC8507286; doi:10.18632/aging.203534)
Supplement: Supplementary Table 1 [file aging-13-203534-s001.docx]

**Supplementary Table 1. Association of ASXL1 expression and clinicopathological parameters in TCGA ACC cohort.**

|  | **TCGA ACC (Parametric)** | | |
| --- | --- | --- | --- |
|  | ***N*** | Mean | SD |
| **Clinical Stage** |  |  |  |
| I | 9 | -0.241 | 0.852 |
| II | 37 | 0.136 | 0.845 |
| III | 16 | -0.481 | 1.146 |
| IV | 15 | 0.355 | 1.218 |
| *P value* |  | 0.084 | |
| **Atypical Mitotic Figures** |  |  |  |
| Present | 31 | -0.119 | 1.124 |
| Absent | 29 | 0.104 | 0.832 |
| *P value* |  | 0.389 | |
| **Capsular invasion** |  |  |  |
| Present | 42 | 0.038 | 0.948 |
| Absent | 30 | 0.034 | 1.037 |
| *P value* |  | 0.988 | |
| **Clinical Status 3 Mo Post-Op** |  |  |  |
| Present | 18 | 0.304 | 1.356 |
| Absent | 54 | -0.118 | 0.908 |
| *P value* |  | 0.139 | |
| **Distant Metastasis** |  |  |  |
| M0 | 62 | -0.078 | 0.956 |
| M1 | 15 | 0.355 | 1.218 |
| *P value* |  | 0.140 | |
| **Cytoplasm Presence Less Than Equal 25** |  |  |  |
| Present | 50 | 0.017 | 1.031 |
| Absent | 10 | -0.152 | 0.790 |
| *P value* |  | 0.628 | |
| **Diffuse Architecture** |  |  |  |
| Present | 42 | -0.031 | 1.033 |
| Absent | 19 | 0.045 | 0.890 |
| *P value* |  | 0.782 | |
| **Ethnicity Category** |  |  |  |
| White | 66 | -0.008 | 1.070 |
| Not White | 11 | -0.001 | 0.655 |
| *P value* |  | 0.984 | |
| Latino | 7 | -0.152 | 1.244 |
| Not Latino | 30 | 0.142 | 1.197 |
| *P value* |  | 0.564 | |
| **Excessive Hormone** |  |  |  |
| Present | 48 | 0.013 | 1.184 |
| Absent | 26 | -0.063 | 0.643 |
| *P value* |  | 0.761 | |
| **Primary Tumor Laterality** |  |  |  |
| Left | 45 | 0.161 | 1.027 |
| Right | 34 | -0.213 | 0.952 |
| *P value* |  | 0.102 | |
| **Metastatic Sites** |  |  |  |
| Lung | 8 | 0.393 | 1.549 |
| Not Lung | 9 | -0.003 | 0.980 |
| *P value* |  | 0.533 | |
| Liver | 8 | 0.077 | 0.976 |
| Not Liver | 9 | 0.279 | 1.512 |
| *P value* |  | 0.752 | |
| **Mitotic Rate > 5/50 HPF** |  |  |  |
| Present | 35 | 0.153 | 1.113 |
| Absent | 29 | 0.060 | 0.870 |
| *P value* |  | 0.717 | |
| **Necrosis** |  |  |  |
| Present | 57 | 0.088 | 1.055 |
| Absent | 17 | -0.042 | 0.655 |
| *P value* |  | 0.631 | |
| **Nuclear Grade** |  |  |  |
| I-II | 13 | 0.048692308 | 0.838054594 |
| III-IV | 49 | 0.002273469 | 1.030367712 |
| *P value* |  | 0.882 | |
| **Lymph node** |  |  |  |
| N0 | 68 | -0.003 | 0.917 |
| N1 | 9 | 0.080 | 1.671 |
| *P value* |  | 0.819 | |
| **T stage** |  |  |  |
| T1 | 8 | -0.240 | 0.911 |
| T2 | 41 | 0.117 | 1.033 |
| T3 | 8 | 0.024 | 1.195 |
| T4 | 18 | -0.170 | 1.033 |
| *P value* |  | 0.849 | |
| **Adjuvant therapy** |  |  |  |
| Yes | 51 | -0.066 | 1.090 |
| No | 25 | 0.179 | 0.869 |
| *P value* |  | 0.331 | |
| **Recurrence on mitotane** |  |  |  |
| Present | 4 | -0.495 | 0.805 |
| Absent | 16 | -0.350 | 0.977 |
| *P value* |  | 0.788 | |
| **Adjuvant RT** |  |  |  |
| Present | 59 | -0.015 | 1.082 |
| Absent | 16 | 0.230 | 0.683 |
| *P value* |  | 0.393 | |
| **Surgical margin** |  |  |  |
| R0 | 55 | -0.070 | 0.911 |
| R1 | 6 | -0.563 | 1.101 |
| R2 | 8 | 1.020 | 1.012 |
| *P value* |  | 0.004 | |
| *Post-hoc P value* |  | R0 vs. R2 | 0.009 |
| *Post-hoc P value* |  | R1 vs. R2 | 0.007 |
| **Gender** |  |  |  |
| F | 48 | 0.040 | 1.114 |
| M | 31 | -0.061 | 0.827 |
| *P value* |  | 0.666 | |
| **Sinusoid Invasion** |  |  |  |
| Present | 9 | 0.307 | 1.530 |
| Absent | 19 | -0.045 | 0.831 |
| *P value* |  | 0.433 | |
| **Primary Therapy Outcome** |  |  |  |
| CR | 34 | -0.234 | 0.942 |
| PD | 16 | 0.396 | 1.385 |
| *P value* |  | 0.065 | |
| **Neoplasm Status** |  |  |  |
| With Tumor | 35 | 0.227 | 1.182 |
| Tumor free | 40 | -0.171 | 0.849 |
| *P value* |  | 0.095 | |
| **Weiss Venous Invasion** |  |  |  |
| Present | 31 | 0.188 | 1.171 |
| Absent | 39 | -0.036 | 0.814 |
| *P value* |  | 0.350 | |
